# Sub-MICs of *Carum copticum* and *Thymus vulgaris* influence virulence factors and biofilm formation in *Candida* spp

**DOI:** 10.1186/1472-6882-14-337

**Published:** 2014-09-15

**Authors:** Mohd SA Khan, Iqbal Ahmad, Swaranjit S Cameotra, Francien Botha

**Affiliations:** Phytomedicine Programme, Department of Paraclinical Sciences, University of Pretoria, Pretoria, 0110 South Africa; Department of Agricultural Microbiology, Aligarh Muslim University, Aligarh, 202002 India; Environmental Biotechnology and Microbial Biochemistry Laboratory, Institute of Microbial Technology, Chandigarh, 160036 India

## Abstract

**Background:**

Emergence of drug-resistant strains of *Candida* and inefficiency of conventional antifungal therapy has necessitated the search for alternative and new antifungal agents. Inhibition of virulence and biofilm are the potential drug targets. In this study, the oils of *Carum copticum*, *Thymus vulgaris* and their major active compound thymol as revealed by Gas chromatography and gas chromatography–mass spectrometry (GC-GC/MS) analysis were tested for their inhibitory activity against growth to determine sub-MIC values against 27 drug-resistant strains of *Candida* spp.

**Methods:**

Brothmacrodilution method was used for determination of MIC of test oils against *Candida* strains. The spectrophotometric methods were used for detection and inhibition assays for virulence factors in *Candida* spp. Light and electron microscopy was performed to observe morphological effects of oils on biofilms. GC-GC/MS were used to evaluate the major active compounds of test oils.

**Results:**

Virulence factors like proteinase and haemolysin were detected in 18 strains, both in solid and liquid media. A 70% of the test strains exhibited hydrophobicity and formed moderate to strong biofilms (OD_280_ 0.5- > 1.0). Test oils exhibited MICs in the range of 45–360 μg.mL^−1^ against the majority of test strains. All the oils at 0.25× and 0.5× MICs induced >70% reduction in the cell surface hydrophobicity, proteinase and haemolysin production. At 0.5× MIC, thymol and *T. vulgaris* were most inhibitory against biofilm formation. At sub-MICs electron microscopic studies revealed the deformity of complex structures of biofilms formed and cell membranes appeared to be the target site of these agents.

**Conclusions:**

Therefore, our findings have highlighted the concentration dependent activity of oils of *C. copticum* and *T. vulgaris* against virulence factors and biofilms in proteinase and haemolysin producing drug-resistant strains of *Candida* spp. The above activities of test oils are supposed to be mainly contributed due to their major active compound thymol. Further mechanism involving anti-proteinase, anti-haemolysin and anti-biofilm activities of these oils and compounds are to be explored for possible exploitation in combating *Candida* infections.

## Background

*Candida* spp cause infections of immuno-competent individuals and, are frequently life-threatening, in particular in immuno-compromised individuals, whose numbers are constantly increasing due to organ transplant, chemotherapy, or, AIDS and Hepatitis C [1]. *Candida* spp now ranks as fourth most common cause of nosocomial bloodstream infection in the United States and the attributable mortality rate is 35% [2]. About 70% of women experience vaginal infections caused by *Candida* spp and 20% of them suffered from recurrence [3]. *Candida albicans* accounts for the majority of cases with candidiasis, but an increasing number of infections due to non-*albicans* spp. have been reported [4]. The most commonly isolated non-*albicans Candida* are *C. glabrata* (causing 3%-35% of all candidemias), followed by *C. tropicalis*, *C. parapsilosis*, *C. krusei*, and other *Candida* spp [5, 6]. Moreover, the majority of such manifestations of candidiasis are associated in one way or another with the formation of *Candida* biofilms on the surfaces of inert or biological surfaces [7]. Biofilm cells are notoriously resistant to antimicrobial agents and withstand host immune defenses [8].

Many of the polyenes and azoles used to treat such infections have several problems namely undesirable side effects, rapid development of drug-resistance and inefficacy against biofilm forming pathogens [9]. This scenario has exacerbated the need for alternative antifungal therapy and search for new and better agents that target fundamental biological processes and/or pathogenic determinants. It is expected that compounds with anti-virulence and antibiofilm activities may reduce or interfere with the production of one or more virulence factors and tolerance to drugs at lower doses. This will attenuate pathogenicity of microorganism without producing killing pressure and therefore development of resistance could be overcome. Further biofilm inhibition by the compound will also reduce the persistence and increased tolerance to drugs.

In the past decade interest in natural products has increased, and medicinal plants have been investigated for various biological activities and therapeutic potentials [10–13]. Oils of *Carum copticum* and *Thymus vulgaris* have been shown to exhibit antifungal activities against pathogenic isolates of *Candida* spp [14–21]. However little or no information is available on anti-infective properties of these oils at non-growth inhibitory concentrations i.e. sub-MICs. Therefore, one way to demonstrate these oils to be effective antipathogenic agents is to check their ability to arrest the production of extracellular enzymatic virulence factors in *Candida* spp that assist the pathogen to colonize host tissues, cause disease, and overcome host defenses [22].

Multiple characteristics of *C. albicans* have been proposed as virulence traits including the phenotypic variability, germination, adherence to inert and biological substrates, cell-surface hydrophobicity and production of secreted hydrolytic enzymes such as aspartyl proteinases, phospholipases and haemolysin [23–26]. On the other hand it has been speculated that biofilms account for as much as 65% of all microbial infections [27]. Since, ability of a pathogen to form biofilm is intimately associated with its adhering potential (cell surface hydrophobicity) and production of virulence factors; we assumed that essential oils or compounds exhibiting anti-virulence activity might be effective against *Candida* biofilms too.

Therefore, first we attempted to assess the production of virulence factors (proteinase and haemolysins) and also cell surface hydrophobicity and biofilm formation in *Candida* spp obtained from various clinical origin. Secondly, sub-MICs of the oils of *C. copticum* and *T. vulgaris* and their major active compound thymol were determined to evaluate in vitro efficacy in influencing virulence and biofilm formation by *Candida* spp. Fluconazole was used as control drug in the study.

## Methods

### Organisms and media

In this study, 23 isolates of clinical origin and 4 reference strains of *Candida* spp exhibiting varying level of resistance to antifungal drugs with MIC of fluconazole ranging from 128 to 256 μg.mL^−1^ [28] were included. The clinical isolates of *C. albicans* (CA01-18), *C. glabrata* (01,02), *C. krusei* 01 and *C. tropicalis* (01,02) were isolated from patients with vaginitis, urinary tract infections and candidemia attending the Jawaharlal Nehru Medical College and Hospital, Aligarh Muslim University, Aligarh. All patients gave written informed consent and the use of these isolates in our study was approved by the institutional ethics committee of the Jawaharlal Nehru Medical College and Hospital, Aligarh Muslim University. *Candida albicans* NRRLY12983 was kindly provided by the fungal culture collection at Agricultural Research Service, USDA, Peoria, USA. The reference strain *C. albicans* SC5314 exhibiting production of various virulence factors was provided by Prof. Rajendra Prasad, Jawaharlal Nehru University, New Delhi. Strains *C. albicans* MTCC183 and *C. glabrata* MTCC3019 were purchased from the Microbial Type Culture Collection, Institute of Microbial Technology, Chandigarh, India. The test strains were maintained on Sabouraud dextrose agar (SDA) slants at 4°C and sub-cultured in the Sabouraud dextrose broth (SDB) prior to use.

### Plant essential oils and drugs

Oils of *Carum copticum* and *Thymus vulgaris* were purchased from Aroma Sales Corporation, New Delhi, India. Whereas, thymol (minimum assay 99%) and drug powder of fluconazole were purchased from Hi-media Laboratories, India and Pfizer Co, Mumbai, India, respectively. Stock solutions of fluconazole was prepared in dimethyl sulphoxide (DMSO) at a concentration of 25 mg.mL^−1^ and stored at −20°C until used. Essential oils were diluted ten-fold in 1% DMSO and used in the assays.

### Detection of virulence factors in isolates of *Candida*spp

#### Proteinase assay

Production of extracellular proteinase was assessed by the method of Aoki *et al.* [29]. using BSA agar plates. Briefly, 60 mL of a solution containing MgSO_4_.7H_2_O 0.04 g, K_2_HPO_4_ 0.5 g, NaCl 0.2 g, yeast extract 0.2 g, glucose 4.0 g and BSA (Hi-Media) 0.5 g; was prepared and the pH was adjusted to 3.5 with 1 N HCL. This solution was filter sterilized and mixed with 140 ml of autoclaved melted agar to prepare the BSA agar plates. Amounts of 10 μL of yeast suspension (1.0 × 10^6^ cfu.mL^−1^) were spot inoculated on the BSA agar plates and incubated at 37°C for 5 days. After incubation, the plates were stained with 0.1% amido black dye and destained with 15% acetic acid, and the clear zone was measured. Proteinase activity (pZ values) was calculated in terms of the ratio of the diameter of the colony to the total diameter of colony plus zone of solubilization.

The proteinase production in liquid medium was determined using the modified method of Kuriyama *et al.* [30]. Briefly, 50 ml Erlenmeyer flasks containing 10 ml of induction medium as described by MacDonald & Odd [31] was inoculated with 100 μl of *Candida* cells (1.0 × 10^6^ cfu ml^−1^) and incubated at 26°C for 7 days at 160 rpm. The broth culture was centrifuged at 5000 rpm for 30 min to obtain supernatant. Furthermore, 200 μL of supernatant was mixed with 800 μL of substrate [1% (w/v) BSA in 0.025 M sodium citrate buffer, pH 3.2] and incubated at 37°C for 3 h. The reaction was halted by the addition of 2.0 mL of 5% trichloroacetic acid (TCA) resulting in precipitation of undigested BSA. Tubes were kept at ice for 30 min and centrifuged at 2000 *g* for 20 min. Proteolysis was determined by measuring the absorbance of soluble peptides at 280_nm_. A control was run by mixing substrate to supernatant and immediately reaction was stopped by adding TCA. The absorbance value of control was subtracted from test samples to obtain values for enzyme activity.

### Haemolysis assay

Haemolysin production was evaluated using a modified method of Luo *et al.* [32]. The blood agar plates were prepared by adding 7 ml of fresh sheep blood suspended in sterile phosphate buffer saline (PBS) to 100 mL of SDA supplemented with 3% (w/v) glucose. Amounts of 10 μl of yeast suspension (1.0 × 10^6^ cfu.mL^−1^) were spot inoculated on the blood agar plates and incubated at 37°C in 5% CO_2_ for 48 h. After incubation, plates were scored for the presence of transluscent ring and or a greenish black halo circumscribing the inoculum growth. Haemolysin activity (pZ values) was calculated in terms of the ratio of the diameter of the colony to the total diameter of colony plus zone of halo.

The haemolysin production in liquid medium was determined using the method of Manns *et al.* [33] with some modifications. The erythrocytes were harvested by centrifugation of freshly obtained sheep blood for 10 min at 3000 rpm, and washed three times in PBS. To the pellet, PBS was added to yield a 10% (v/v) erythrocytes/PBS suspension. The 10% suspension was then diluted 10 times in PBS. *Candida* cells were grown in SDB [8% (w/v) glucose] for 4 days at 37°C. The broth cultures were centrifuged at 5000 *g* for 30 min to obtain supernatant. Furthermore, the culture supernatant and RBC suspension were mixed in 1:1 ratio and incubated at 37°C for 4 h. Triton X-100 [0.1% (v/v) in PBS] was used as a positive control whereas 1% DMSO and PBS were used as negative controls. Tubes were centrifuged at 2000 *g* for 10 min and the absorbances of supernatants were read at 540_nm_. The percentage of haemolysis was calculated as: [{(A − B)/(C − B)} × 100]. Where, A and B are the absorbance values of supernatant from the test sample and PBS (solvent control) respectively and C is the absorbance value of supernatant from the sample after 100% lysis.

### Detection of cell surface hydrophobicity and biofilm formation in the isolates of *Candida*spp

#### Cell surface hydrophobicity

The method of microbial adhesion to hydrocarbons (MATH) as described by Rosenberg *et al.* [34] was used with some modifications to determine the %CSH of *Candida* cells. Briefly, *Candida* cells were grown on SDA plates amended with 0.5 × and 0.25 × MICs of test agents at 25°C for 48 h. *Candida* cells grown on SDA plates without amendment of test agents were considered as untreated control. Yeast suspensions were made in PBS (A_520_ of 0.400) and allowed to contact with xylene in 5:1 ratio. The mixtures were vortexed for 1 min at room temperature and then allowed to separate the two phases for 10 min. The absorbance of aqueous phase was read at 520 nm. The %CSH was calculated as follows; %CSH = [(A_i_ − A_f_)/A_i_] × 100. Where A_i_ and A_f_ are the absorbance values of aqueous yeast suspension at 520 nm before and after contacting yeast suspension to the organic phase.

### Biofilm formation

The strains were evaluated by 96 well microtiter plates (HiMedia, Laboratories, Mumbai, India) based XTT reduction assay for their ability to form biofilms using the method of Ramage *et al.* [35] with some modifications. Briefly, *Candida* cells were grown in SDB (glucose 8% [w/v]) at 37°C for 24 h. Cells were harvested and re-suspended in RPMI 1640 medium with L-glutamine but without bicarbonate and buffered to pH 7.0 with MOPS to a cell density of 1.5 × 10^6^ cfu.mL^−1^. Biofilms were formed by adding 100 μL of this standardized cell suspension to wells of microtiter plates and incubating at 37°C for 48 h. After biofilm formation, the medium was aspirated gently, and non-adherent cells were removed by washing the biofilms three times with sterile PBS. Further biofilm formation was analysed by XTT reduction assay. Briefly, 0.091 mL of XTT (Sigma, N. Delhi) (1 μg.mL^−1^, prepared in PBS) and 0.009 mL of menadione (Sigma, N. Delhi) (1 mmol.L^−1^, prepared in acetone) was added to each well and incubated in the dark for 4 h. The colorimetric change was measured at 492 nm using a Labsystem Multiskan Ex MTP Reader.

### Light microscopy of biofilms formed

Polyvinyl chloride catheter discs (15 mm diameter) were placed in 6 well tissue culture plates. Wells of the plates were dispensed with 1 mL of RPMI medium and 1 ml of standardized yeast cell suspension was added to each well and incubated at 37°C for 48 h. After the formation of biofilm, medium was discarded and discs were washed three times with sterile PBS and air dried. The discs were visualized under bright field light microscope (Olympus, Japan) at 40 X.

### Gas chromatography and gas chromatography–mass spectrometry analysis of essential oils

The percentage composition of oils and compounds was determined by GC-FID and the compounds were identified by GC-MS. GC analysis was carried out on a Shimadzu 2010 Gas Chromatograph equipped with an FID and 25 m × 0.25 mm × 0.25 μm WCOT column coated with diethylene glycol (AB-Innowax, 7031428, Japan). Injector temperature was set at 270°C and detector at 280°C. Nitrogen was used as a carrier gas at a flow rate of 3.0 ml/min at a column pressure of 74.9 kPa. 0.2 μL of sample were injected into column with a split ratio of 90.0. The linear temperature program of 60°C to 230°C set at a rate of 3°C min^−1^ with hold time at 230°C for 10 minutes. The samples were then analyzed on the same Shimadzu instrument fitted with the same column and following the same temperature program as above. MS parameters used were: ionisation voltage (EI) 70 eV, peak width 2 s, mass range 40–600 amu and detector voltage 1.5 V. Results were based on GC-FID. Peak identification was carried out by comparison of the mass spectra with database of NIST05, NBS75K and Wiley 8 libraries. Identification of compounds was confirmed by comparison of their relative retention indices relative to (C8–C22) n-alkanes with literature data or authentic compounds [36–38].

### Determination of sub-MICs essential oils against the strains of *Candida*spp

For MIC determination of essential oils, 10 μL of yeast suspension (0.5 McFarland) was added to 1 mL SDB containing serially diluted test oils along with 0.1% (v/v) Tween 80 and incubated at 37°C for 24 h at 28 ± 2°C. MIC was defined as the lowest concentration that inhibited visible growth.

### Determination of viability of *Candida*strains at sub-MICs of oils

The test strains at a cell density of 1.0 × 10^6^ cfu.mL^−1^ were inoculated in to test tubes containing 10 ml of SDB amended with sub-MICs (0.5 × and 0.25 × MICs) of test agents for 24 h at 37°C. A control was run without test agents but containing yeast inoculum at equal cell density. Viable counts were obtained from the test and control solutions by plating 100 μL of 10-fold serial dilutions onto SDA plates and incubating at 37°C for 24 h.

### Inhibition of virulence factors by essential oils at sub-MICs

#### Inhibition of proteinase production

Briefly, 50 ml Erlenmeyer flasks containing10 ml of induction medium as described by MacDonald and Odds [31] with and without sub-MICs (0.5 × and 0.25 × MIC) of test agents were inoculated with 100 μL of *Candida* cells (1.0 × 10^6^ cfu.mL^−1^) and incubated at 26°C for 7 days at 160 rpm. The inhibition of proteinase production by oils was determined using the modified method of Kuriyama *et al.* [30] as mentioned earlier. The absorbance value of untreated control was subtracted from treated samples to obtain values for enzyme activity. Each experiment was conducted three times with three replicates per experiment and data are presented as percent reduction of absorbance values in treated sample over untreated control.

#### Inhibition of haemolysin production

*Candida* cells were grown in SDB [8% (w/v) glucose] containing 0.5 × and 0.25 × MIC of test agents for 4 days at 37°C. The solution without test agents was considered as untreated control. Further, the inhibition of haemolysin production by oils was carried out using the method of Manns *et al.* [33] with some modifications as mentioned earlier. Each experiment was conducted three times with three replicates per experiment and mean absorbance values were used to calculate the percent reduction of haemolysin in treated samples over untreated controls.

### Inhibition of biofilm formation by essential oils at sub-MICs

#### Effect of oils on biofilm formation

The effect of different sub-MICs of oils and drugs (0.5 × MIC, 0.25 × MIC) on the ability of *Candida* cells to form biofilm was determined by the modified method of Ramage *et al.* [35] with some modifications. Volumes of 0.1 mL of test agents (2 × final concentrations) in RPMI 1640 medium was added to each well of microtiter plates. Subsequently, 0.1 mL of standardized yeast cell suspension was added and plates were incubated at 37°C for 48 h. Antifungal agent-free wells served as positive controls for biofilm growth. After incubation, the medium and non-adherent cells were removed from wells and washed three times with sterile PBS. Further biofilm formation was analysed by XTT reduction assay. Absorbance values were used to measure the inhibition of biofilm formation as follows: (mean OD_492_ of treated well/mean OD_492_ of untreated control well) × 100.

### Scanning electron microscopy of biofilm cells formed in the presence of oils

For examination by SEM, *Candida* biofilm cells were grown on catheter discs in the presence of 0.5 × and 0.25 × MICs of test agents for 48 h at 37°C as described in the preceding section. Biofilms grown in the absence of test agents served as control. After washing with PBS, discs containing biofilms were placed in a fixative solution of 5% glutaraldehyde in cacodylate buffer in a graded series of ethanol, immersed in hexamethyldisilazane, and finally air dried overnight at room temperature.

### Transmission electron microscopy of planktonic cells

Structural changes produced by test compounds towards fungal cell were evaluated using transmission electron microscopy. Briefly, 10 mL of SDB treated with 0.5 × and 0.25 × MICs of test agents was inoculated with 100 μL of cell suspension (0.5 McFarland) and incubated at 37°C for 48 h at 120 rpm. Control sample did not receive treatment. The obtained cell pellets were fixed with 2.5% glutaraldehyde in 0.1 M cacodylate buffer (pH 7.2) for 24 h at room temperature. Post fixation was carried out in 1% osmium tetroxide in cacodylate buffer. Samples were dehydrated in acetone and embedded in epon. Ultra thin sections were stained with 12.5% alcoholic uranyl acetate and viewed under Morgagni 268D transmission electron microscope at 80 kv.

### Statistical analysis

All the experiments were performed three times with three replicates per experiment and data are expressed as mean ± standard deviation. Statistical significance of the differences was determined by the one way ANOVA test using Minitab (V.11.0 for Windows). Reduction in %CSH in test strains in the presence of oils was compared to untreated control by one way ANOVA using Duncan’s method. Similarly, difference in log cfu/ml of treated samples (0.5 × and 0.25 × MICs) was compared to untreated control. *P*-values of < 0.05 were considered as statistically significant.

## Results

### Detection of proteinase and haemolysin production

**Figure 1:**
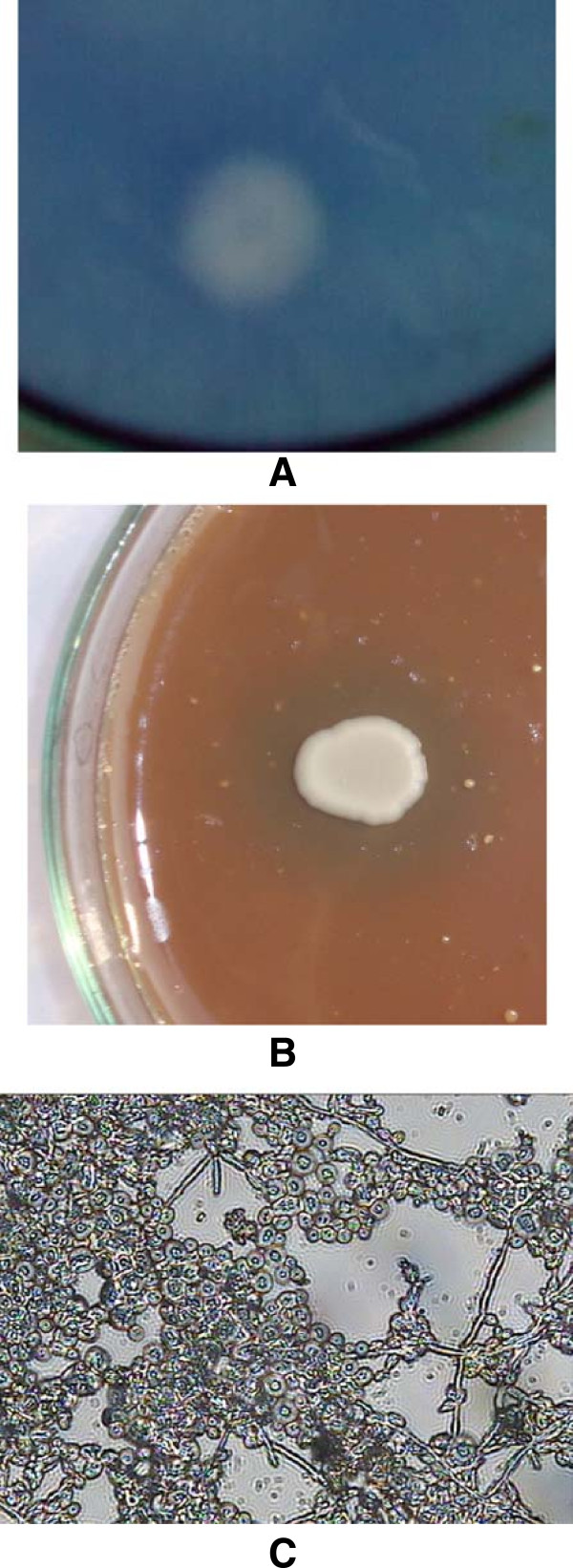
**Detection of extracellular expression of virulence factors in the strains of** ***Candida*** **spp in solid media. (A)** Proteinase production in *C. albicans* 04. **(B)** Haemolysin production in *C. albicans* 02. **(C)** Biofilm formation by *C. albicans* 04 on catheter discs in 48 h, under 40 × light microscope (mature biofilms exhibiting dense matrix, hyphae and embedded yeast cells).

As presented in Table 1 and Figure 1A,B, among the test strains of *C. albicans* 71% were positive for proteinase production and 52% were for haemolysin production on solid medium. Among the strains of non-albicans *Candida* (NAC) production of proteinase and haemolysin was recorded in 3 different strains.

**Table 1:** **Detection of biofilm formation and extracellular production of virulence factors in the strains of** ***Candida*** **spp**

Test strains	Clinical condition	Resistance pattern	% CSH	Biofilm formation (OD_490_)*	Virulence factors production			
					Solid medium		Liquid medium	
					Activity inde × (pZ)			
					Proteinase	Haemolysin	Proteinase (OD_280_)#	% haemolysis
***C. albicans***								
*C. albicans* 01	Candidemia	KTZ, ICZ, FLZ	41.30 ± 3.75	0.49 ± 0.04	0.50 ± 0.04	0.63 ± 0.05	1.23 ± 0.11	41.52
*C. albicans* 02	-Do-	KTZ, ICZ, FLZ	ND	0.05 ± 0.01	0.78 ± 0.04	0.60 ± 0.05	0.45 ± 0.02	29.10
*C. albicans* 03	-Do-	KTZ, ICZ, FLZ	62.11 ± 2.31	0.59 ± 0.02	0.55 ± 0.03	1.0 ± 0.00	1.35 ± 0.13	ND
*C. albicans* 04	-Do-	KTZ, ICZ, FLZ	61.75 ± 2.85	1.36 ± 0.66	0.80 ± 0.05	1.0 ± 0.00	0.42 ± 0.04	ND
*C. albicans* 05	Urine	AMB, KTZ, ICZ, FLZ	59.78 ± 4.31	1.0 ± 0.08	1.0 ± 0.00	0.68 ± 0.05	Nil	74.68
*C. albicans* 06	-Do-	AMB, KTZ, ICZ, FLZ	55.43 ± 3.38	1.15 ± 0.10	0.60 ± 0.04	1.0 ± 0.00	0.95 ± 0.04	ND
*C. albicans* 07	-Do-	AMB, KTZ, ICZ, FLZ	66.21 ± 2.97	0.60 ± 0.03	0.71 ± 0.06	1.0 ± 0.00	0.44 ± 0.03	ND
*C. albicans* 08	-Do-	KTZ, ICZ, FLZ	ND	0.28 ± 0.04	0.52 ± 0.05	1.0 ± 0.00	1.18 ± 0.14	ND
*C. albicans* 09	-Do-	AMB, KTZ, ICZ, FLZ	55.10 ± 2.75	1.12 ± 0.09	0.77 ± 0.04	0.83 ± 0.05	0.85 ± 0.06	25.84
*C. albicans* 10	Vaginitis	AMB, KTZ, ICZ, FLZ	ND	0.05 ± 0.01	0.66 ± 0.05	1.0 ± 0.00	1.37 ± 0.22	ND
*C. albicans* 11	-Do-	AMB, KTZ, ICZ, FLZ	ND	0.2650.03	0.75 ± 0.04	0.63 ± 0.04	0.61 ± 0.05	37.33
*C. albicans* 12	-Do-	AMB, KTZ, ICZ, FLZ	ND	0.35 ± 0.05	0.71 ± 0.04	1.0 ± 0.00	1.00 ± 0.09	ND
*C. albicans* 13	-Do-	AMB, KTZ, ICZ, FLZ	25.78 ± 2.98	0.67 ± 0.08	0.78 ± 0.05	1.0 ± 0.00	1.15 ± 0.10	ND
*C. albicans* 14	-Do-	AMB, KTZ, ICZ, FLZ	33.45 ± 3.71	0.62 ± 0.05	0.73 ± 0.06	1.0 ± 0.00	1.18 ± 0.10	ND
*C. albicans* 15	-Do-	AMB, KTZ, ICZ, FLZ	44.59 ± 4.97	0.55 ± 0.04	1.0 ± 0.00	1.0 ± 0.00	Nil	ND
*C. albicans* 16	-Do-	AMB, KTZ, ICZ, FLZ	24.76 ± 2.38	0.58 ± 0.01	1.0 ± 0.00	0.65 ± 0.05	Nil	83.18
*C. albicans* 17	-Do-	AMB, KTZ, ICZ, FLZ	40.90 ± 3.36	0.78 ± 0.02	1.0 ± 0.00	0.64 ± 0.04	Nil	81.25
*C. albicans* 18	-Do-	AMB, KTZ, ICZ, FLZ	53.45 ± 4.97	1.32 ± 0.07	1.0 ± 0.00	0.68 ± 0.05	Nil	91.45
*C. albicans* NRRLY12983	Reference strain	KTZ, ICZ, FLZ	25.78 ± .17	1.15 ± 0.10	0.50 ± 0.04	0.73 ± 0.06	1.21 ± 0.10	24.32
*C. albicans* MTCC183	-Do-	KTZ, ICZ, FLZ	35.60 ± 1.76	0.60 ± 0.03	1.0 ± 0.00	0.82 ± 0.06	1.35 ± 0.12	24.93
*C. albicans* SC5314	-Do-	-	63.28 ± 5.18	1.09 ± 0.01	0.58 ± 0.05	0.55 ± 0.06	1.06 ± 0.09	74.45
**Non albicans** ***-Candida***								
*C. glabrata* 01	Vaginitis	KTC, ICZ, FLZ	65.39 ± 6.1	0.05 ± 0.01	1.0 ± 0.00	1.0 ± 0.00	0.20 ± 0.05	ND
*C. glabrata* 02	-Do-	AMB, KTZ, ICZ, FLZ	ND	0.04 ± 0.09	1.0 ± 0.00	1.0 ± 0.00	Nil	15.96
*C. glabrata* MTCC3019	Reference strain	KTC, ICZ, FLZ	ND	0.05 ± 0.09	1.0 ± 0.00	1.0 ± 0.00	Nil	ND
*C. krusei* 01	Vaginitis	AMB, KTZ, ICZ, FLZ	41.22 ± 3.38	0.05 ± 0.01	0.55 ± 0.04	0.61 ± 0.04	Nil	28.95
*C. tropicalis* 01	Candidemia	ICZ, FLZ	62.99 ± 5.10	0.14 ± 0.01	0.56 ± 0.04	0.63 ± 0.05	0.98 ± 0.07	60.42
*C. tropicalis* 02	Vaginitis	ICZ, FLZ	ND	0.06 ± 0.02	0.65 ± 0.05	0.66 ± 0.05	0.57 ± 0.06	34.27

pZ = (zone of diameter of colony)/(zone of diameter of colony + zone of solubilization or precipitation around the colony).

pZ = 1; indicates no activity.

*Biofilm formation was considered as Nil for OD_490_ < 0.1.

OD_490_ (0.1-0.5); weak biofilm, OD_490_ (>0.5-1.0); moderate biofilm, OD_490_ (>1.0); strong biofilms.

#Proteinase activity was considered as Nil for OD_280_ < 0.1.

AMB; amphotericin B, FLZ; fluconazole, ICZ; Itraconazole, KTZ; ketoconazole.

ND; not detected.

Production of proteinase and haemolysin in liquid medium among the strains of *Candida* spp is depicted in Table 1. Production of proteinase was recorded in 75% of the test strains of *C. albicans*. Seven strains from different clinical origin and three reference strains exhibited higher proteolytic activity with absorbance values (>1.0). Haemolysin production was recorded in 52% of the *C. albicans* strains. Among the NACs, four test strains exhibited haemolysin production in the range of 15.96 to 60.42%. All the strains producing protease on solid medium could also produce in liquid medium except three strains *C. albicans* MTCC12983, *C. glabrata* 01 and *C. tropicalis* 02. Similar trend was also observed in the production of haemolysin on solid and liquid medium.

### Detection of cell surface hydrophobicity of *Candida*cells and their ability to form biofilms

As evident from Table 1 76% of the test strains of *C. albicans* were positive for CSH (24.76 to 66.21% CSH) as well as proteinase production (A_260_ nm 0.45 to 1.61). Among the NACs three strains were hydrophobic with %CSH ranging from 41.22 to 65.39.

Out of 18 clinical and three reference strains of *C. albicans*, 13 strains formed moderate to strong biofilms. Three strains produced weak biofilms and two strains could not form biofilms (Table 1 and Figure 1C). Reference strains *C. albicans* NRRLY12983 and *C. albicans* SC5314 formed strong biofilms with absorbance values of >1.0. Among the 6 non-albicans *Candida*, only *C. tropicalis* 01 formed weak biofilm and others were negative. Among the test strains, non-hydrophobic strains (viz. *C. albicans* 02, 08, 10–12, *C. glabrata* 02, *C. glabrata* MTCC3019 and *C. tropicalis* 02) were either non-biofilm or weak biofilm formers.

### GC-GC/MS analysis

GC and GC-MS analysis of essential oils revealed the presence of various major and minor compounds. Main *C. copticum* oil components were ρ-cymene (33.67%), thymol (22.82%) and γ-terpinene (21.61%). Thymol was the main constituent (44.71%) of *T. vulgaris* followed by γ-terpinene (26.01%) and α-cymene (21.22%) (Tables 2 and 3).

**Table 2:** **Major active compounds of** ***C. copticum*** **as identified by GC/GC-MS analysis**

Peak no.	Retention indices	Compound identified	% area
1	939	α-pinene	4.5
4	980	β-pinene	12.3
7	992	β-myrcene	2.6
10	1033	α-limonine	0.6
12	1061	γ-terpinene	21.6
13	1017	ρ-cimene	33.8
15	1096	α-dimethyl styrene	0.8
17	1109	limonene o × ide	0.5
22	1288	thymol	22.8

**Table 3:** **Major active compounds of** ***T. vulgaris*** **as identified by GC/GC-MS analysis**

Peak no.	Retention indices	Compound identified	% area
1	936	α-pinene	1.1
3	977	β-pinene	0.5
5	1033	α-limonine	0.5
7	1061	γ-terpinene	26.0
8	1023	α- cimene	21.2
11	1078	Z,6-dimethyl-3,5,7-octriene-olstyrene	0.5
17	1288	thymol	44.7
19	1318	carvacrol	0.9
22	1514	1,4-inden-1-one	0.6
24	1564	3-pentene-2-one	1.1

### Susceptibility of fungal strains to essential oils and viability at sub-MICs

As evident from Table 4, test oils exhibited MIC value in the range of 45–360 μg/ml against one or other strains. There was no significant (*P* < 0.05) decrease observed in log cfu in the test strains of *C. albicans* at cell density of 1.0 × 10^6^ cfu.mL^−1^ treated with 0.25 × and 0.5 × MICs of test agents compared to untreated control at equal cell density (Table 5).

**Table 4:** **Sensitivity of strains of** ***Candida*** **spp to oils of** ***C. copticum*** **,** ***T. vulgaris*** **and thymol**

Test strains	MIC (μg.mL^−1^)		
	***C. copticum***	***T. vulgaris***	Thymol
***C. albicans***			
*C. albicans* 01	360	180	90
*C. albicans* 02	180	180	90
*C. albicans* 03	180	360	90
*C. albicans* 04	180	360	180
*C. albicans* 05	180	360	90
*C. albicans* 06	360	360	180
*C. albicans* 07	360	90	45
*C. albicans* 08	180	90	45
*C. albicans* 09	360	180	90
*C. albicans* 10	360	90	90
*C. albicans* 11	360	360	90
*C. albicans* 12	90	180	90
*C. albicans* 13	360	360	180
*C. albicans* 14	360	360	180
*C. albicans* 15	360	180	90
*C. albicans* 16	360	360	90
*C. albicans* 17	360	90	45
*C. albicans* 18	180	180	45
*C. albicans* NRRLY12983	180	180	90
*C. albicans* MTCC183	180	90	90
*C. albicans* SC5314	45	90	90
*C. glabrata* 01	180	360	180
*C. glabrata* 02	180	360	90
*C. glabrata* MTCC3019	180	360	90
*C. krusei* 01	360	360	180
*C. tropicalis*01	90	360	90
*C. tropicalis*02	90	360	90

**Table 5:** **Viability assays of strains of** ***Candida*** **spp. e × posed to sub-inhibitory concentrations of oils of** ***C. copticum*** **,** ***T. vulgaris*** **and thymol**

Test agents (sub-MICs)	Cell viability assay (log cfu.mL^−1^at 10^5^dilution)							
	***C. albicans***04	***C. albicans***05	***C. albicans***07	***C. albicans***09	***C. albicans***16	***C. albicans***MTCC183	***C. albicans***SC5314	***C. tropicalis***01
Untreated	8.14 ± 0.02	8.04 ± 0.01	8.12 ± 0.03	8.25 ± 0.01	8.02 ± 0.04	8.19 ± 0.01	8.45 ± 0.03	8.07 ± 0.04
**Essential oils**								
*C. copticum*								
0.25 × MIC	7.16 ± 0.04	7.29 ± 0.07	7.34 ± 0.03	7.26 ± 0.04	7.40 ± 0.06	7.38 ± 0.05	7.60 ± 0.02	7.41 ± 0.04
0.5 × MIC	6.10 ± 0.03	6.39 ± 0.05	6.46 ± 0.04	6.24 ± 0.02	6.35 ± 0.03	6.44 ± 0.04	7.17 ± 0.01	6.98 ± 0.05
*T. vulgaris*								
0.25 × MIC	7.09 ± 0.03	7.24 ± 0.06	7.14 ± 0.05	7.19 ± 0.05	7.37 ± 0.04	7.32 ± 0.05	7.71 ± 0.04	7.52 ± 0.02
0.5 × MIC	6.43 ± 0.06	6.41 ± 0.05	6.39 ± 0.07	6.19 ± 0.04	6.27 ± 0.06	6.44 ± 0.07	7.02 ± 0.04	6.71 ± 0.06
Thymol								
0.25 × MIC	7.11 ± 0.02	7.16 ± 0.04	7.23 ± 0.06	7.22 ± 0.03	7.24 ± 0.06	7.26 ± 0.05	7.44 ± 0.07	7.29 ± 0.03
0.5 × MIC	6.23 ± 0.04	6.54 ± 0.03	6.44 ± 0.03	6.33 ± 0.06	6.23 ± 0.05	6.33 ± 0.05	7.08 ± 0.07	6.45 ± 0.05
**Antifungal drug**								
Fluconazole								
0.25 × MIC	7.08 ± 0.03	7.11 ± 0.06	7.23 ± 0.05	7.16 ± 0.05	7.22 ± 0.04	7.31 ± 0.04	7.14 ± 0.06	7.31 ± 0.05
0.5 × MIC	6.18 ± 0.04	6.45 ± 0.06	6.44 ± 0.05	6.27 ± 0.04	6.15 ± 0.06	6.04 ± 0.05	7.23 ± 0.04	6.88 ± 0.06

Within the columns, untreated means followed by treated means are non-significantly different according to Duncan’s multiple range tests (*P* < 0.05).

### Anti-virulence activity of essential oils against test fungi

Oils of *C.* copticum, *T. vulgaris* and thymol were further assessed for their anti-virulence activity at sub-MICs against the strains *C. albicans* 04, 05, 07, 16, MTCC183, SC5314 and *C. tropicalis* 01. These strains were selected on the basis of being producers of significant amount of proteinase or haemolysin.

### Effect on cell surface hydrophobicity

As evident from Table 6, CSH in test strains was affected in varying capacity when treated with sub-MICs of oils. Test oils at both the 0.5 × and 0.25 × MICs were significantly (*P <* 0.05) effective in reducing the CSH in one or other strains. Control drug fluconazole at tested concentrations could not induce significant reduction in CSH.

**Table 6:** **CSH in the strains of** ***Candida*** **spp. exposed to sub-inhibitory concentrations of oils of** ***C. copticum*** **,** ***T. vulgaris*** **and thymol**

Test agents (Sub-MICs)	Percent cell surface hydrophobicity				
	***C. albicans***04	***C. albicans***07	***C. albicans***MTCC183	***C. albicans***SC5314	***C. tropicalis***01
Untreated control	61.75 ± 3.45^a^	62.74 ± 4.35 ^a^	33.80 ± 3.45^a^	53.28 ± 3.45^a^	60.97 ± 5.34^a^
**Essential oils**					
*C. copticum*					
0.25 × MIC	22.55 ± 2.24^b^	21.16 ± 2.24^b^	22.69 ± 1.91^b^	25.34 ± 2.43^b^	17.31 ± 0.82^b^
0.5 × MIC	15.00 ± 1.67^b,c^	17.87 ± 1.24^b,c^	12.98 ± 1.47^c^	16.72 ± 1.81^c^	6.69 ± 0.45^c^
*T. vulgaris*					
0.25 × MIC	51.37 ± 4.45^b^	33.70 ± 2.34^b^	30.37 ± 2.34^a^	40.27 ± 3.81^b^	43.25 ± 4.16^b^
0.5 × MIC	43.56 ± 3.31^b,c^	24.91 ± 1.81^b,c^	17.05 ± 1.23^b^	20.18 ± 1.67^c^	36.39 ± 2.43^b^
Thymol					
0.25 × MIC	33.17 ± 2.56^a^	28.00 ± 2.67^a^	29.92 ± 2.89^a^	35.81 ± 2.78^a^	34.45 ± 2.67^a^
0.5 × MIC	21.23 ± 1.78^a^	25.11 ± 1.67^b^	16.64 ± 1.78^b^	15.89 ± 1.89^b^	17.89 ± 1.89^b^
**Antifungal drug**					
Fluconazole					
0.25 × MIC	61.68 ± 4.67^a^	43.07 ± 3.89^b^	31.74 ± 3.78^a^	46.89 ± 3.89^a^	51.23 ± 4.78^a^
0.5 × MIC	55.93 ± 3.56^a^	32.35 ± 3.56^b,c^	30.89 ± 3.78^a^	40.10 ± 3.89^b^	43.92 ± 3.89^b^

Within the columns, means values followed by different letters (^a, b, c^) are significantly different according to Duncan’s multiple range tests (*P* < 0.05).

### Effect on production of proteinase and haemolysin

At both of the tested concentrations, test oils were able to reduce the production of proteinase by >70% in test strains (Table 7). Maximum reduction of 96.0% was observed for *T. vulgaris* at 0.5 × MIC against *C. albicans* 04. The test oils at 0.5 × MIC induced >70% reduction in the haemolytic activity of test strains. A maximum reduction of 87.83% was recorded for *C. copticum* at 0.5 × MIC against *C. albicans* 16. *T. vulgaris* at both the tested concentrations induced a reduction of 79.47% against this strain (Table 7).

**Table 7:** **Effects of sub-inhibitory concentrations of oils of** ***C. copticum*** **,** ***T. vulgaris*** **and thymol on proteinase and haemolysin productions in the strains of** ***Candida*** **spp**

Test agents (Sub-MICs)	*Percent reduction in proteinase production over untreated control					**Percent reduction in haemolysin production over untreated control				
	***C. albicans***04	***C. albicans***09	***C. albicans***MTCC183	***C. albicans***SC5314	***C. tropicalis***01	***C. albicans***05	***C. albicans***16	***C. albicans***MTCC183	***C. albicans***SC5314	***C. tropicalis***01
**Essential oils**										
*C. copticum*										
0.25× MIC	89.23 ± 4.56	80.74 ± 3.45	88.50 ± 2.34	70.48 ± 4.56	63.67 ± 2.34	53.90 ± 3.45	74.70 ± 4.56	56.89 ± 2.34	56.89 ± 3.45	58.90 ± 3.67
0.5× MIC	94.80 ± 3.56	84.59 ± 5.67	90.91 ± 5.78	82.45 ± 4.56	82.50 ± 3.45	64.13 ± 4.56	87.83 ± 5.67	77.56 ± 3.33	65.78 ± 3.45	70.89 ± 4.56
*T. vulgaris*										
0.25× MIC	92.54 ± 5.67	86.31 ± 5.67	71.99 ± 3.67	65.78 ± 5.67	75.05 ± 4.56	55.41 ± 3.45	79.47 ± 3.45	48.11 ± 3.45	50.89 ± 4.56	50.46 ± 4.56
0.5× MIC	96.00 ± 2.34	91.45 ± 5.67	80.55 ± 5.67	74.83 ± 4.56	87.34 ± 2.34	71.05 ± 4.56	79.47 ± 4.56	63.16 ± 3.45	59.80 ± 3.45	67.10 ± 3.45
Thymol										
0.25× MIC	95.98 ± 5.78	89.31 ± 4.56	91.99 ± 5.67	90.78 ± 5.67	88.99 ± 4.45	65.41 ± 2.34	85.00 ± 3.45	71.11 ± 4.57	68.89 ± 3.56	66.46 ± 4.56
0.5× MIC	97.00 ± 5.56	93.67 ± 5.67	92.65 ± 4.67	87.97 ± 4.56	90.67 ± 3.34	78.05 ± 4.56	89.47 ± 4.56	83.16 ± 4.34	69.80 ± 3.63	77.10 ± 5.61
**Antifungal drug**										
Fluconazole										
0.25× MIC	50.32 ± 4.47	50.33 ± 4.47	44.13 ± 3.34	43.39 ± 4.56	41.57 ± 4.56	45.32 ± 3.47	49.33 ± 3.47	38.13 ± 4.34	40.39 ± 4.56	40.57 ± 4.56
0.5× MIC	54.23 ± 5.56	57.47 ± 4.57	56.16 ± 4.47	54.80 ± 4.45	54.10 ± 5.33	51.23 ± 4.56	55.47 ± 3.57	46.16 ± 3.47	49.80 ± 3.45	51.10 ± 3.33

*Absorbance at 280 nm for untreated controls were 0.442 ± 0.009 (*C. albicans* 04), 0.865 ± 0.003 (*C. albicans* 09), 1.267 ± 0.006 (*C. albicans* MTCC183), 1.032 ± 0.043 (*C. albicans* SC5314), 1.010 ± 0.007 (*C. tropicalis* 01).

**% haemolysin production in untreated controls were 74.68 ± 3.45 (*C. albicans* 05), 83.18 ± 5.67 (*C. albicans* 16), 24.93 ± 2.67 (*C. albicans* MTCC183), 74.45 ± 4.56 (*C. albicans* SC5314), 60.42 ± 3.45 (*C. tropicalis* 01).

### Inhibition of biofilms

As presented in Table 8, varying level of attenuation in the biofilm formation by *C. albicans* cells was observed in the presence of essential oils. Biofilm formation in both the test strains was reduced maximally by thymol. At the treatment of 0.5 × MIC and 0.25 × MIC of this oil, 6.90 to 8.34% formation of biofilm was recorded in test strains. Whereas fluconazole at 0.5 × MIC led to 48.16% formation of biofilm in *C. albicans* 04. Similar pattern of biofilm formation was also recorded for test oils and drugs against *C. albicans* SC5314.

**Table 8:** **Effects of oils of** ***C. copticum*** **,** ***T. vulgaris*** **and thymol on biofilm formation in drug-resistant and -sensitive strains of** ***C. albicans***

Test agents	Biofilm formation			
	***C. albicans***04		***C. albicans***SC5314	
	0.5 × MIC	0.25 × MIC	0.5 × MIC	0.25 × MIC
**Essential oils**				
*C. copticum*	25.32 ± 2.68	40.08 ± 3.69	28.50 ± 2.41	42.92 ± 2.48
*T. vulgaris*	07.49 ± 0.98	11.70 ± 1.91	14.72 ± 1.05	25.04 ± 2.54
Thymol	07.59 ± 1.69	08.34 ± 1.93	06.90 ± 1.34	07.85 ± 1.18
**Antifungal drug**				
Fluconazole	48.16 ± 0.97	67.61 ± 1.39	48.63 ± 2.24	65.34 ± 3.32

### Scanning electron microscopy of sessile cells

**Figure 2:**
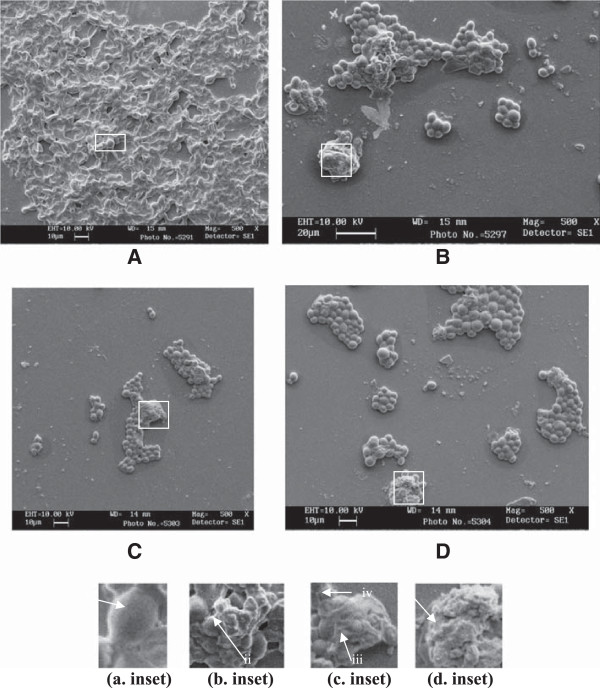
**Scanning electron micrograph of the 48 h biofilm formed in** ***C. albicans*** **04 on catheter discs in the absence and presence of sub-MICs of oils. (A)** Biofilm formed without oils (a. inset) arrow indicates smooth cell membrane of normal cell. **(B)** Biofilm formed in the presence of *C. copticum* at 90 μg.mL^−1^ (b. inset) bursting of cells leading to vesicle formation due to lytic material (ii). **(C)** Biofilm formed in the presence of *T. vulgaris* at 90 μg.mL^−1^ (c. inset) cell membrane shrinkage in sessile cells (iii), empty cells (iv). **(D)** Biofilm formed in the presence of thymol at 90 μg.mL^−1^ (d. inset) lysis of membrane and release of cellular material (v).

Untreated cells resulted in intact biofilm formation with dense matrix and multilayered network of yeast cells and hyphae leading to a compact three dimensional structure (Figure 2A). The treated cells (90 μg ml^−1^ of *C. copticum*) exhibited loosening of cells and disappearance of matrix. Filamentation is inhibited and destruction of cell membrane in biofilm was observed (Figure 2B,C). Untreated sessile cells showed smooth cell membrane (Figure 2a. inset) whereas treatment with oils exhibited shrinkage and lysis in cell membranes of sessile cells (Figure 2b. inset and c. inset). Similar effects were also observed for thymol (Figure 2D, and d. inset).

### Transmission electron microscopy of planktonic cells

**Figure 3:**
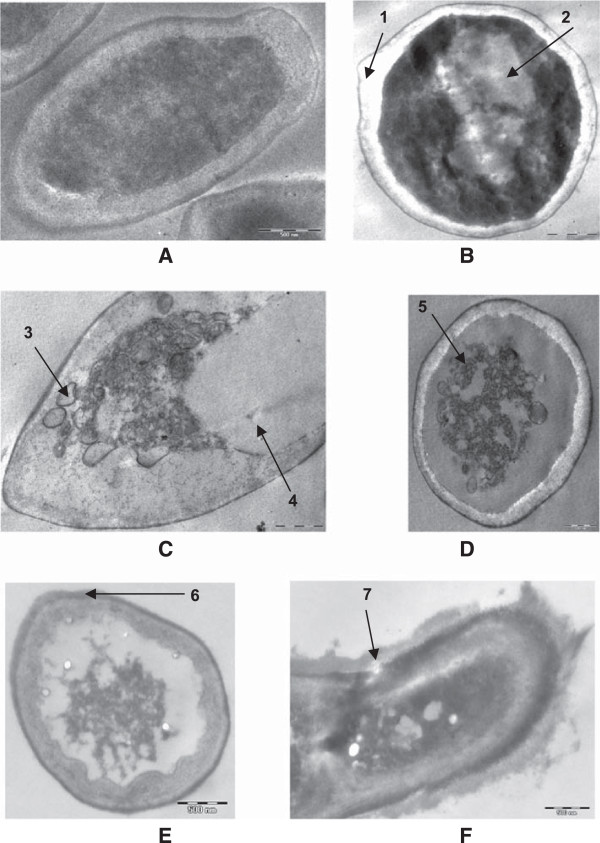
**Transmission electron micrograph of the 48 h grown** ***C. albicans*** **04 cells in the absence and presence of sub-MICs of oils. (A)** Untreated; intact cell wall, cell membranes and other organelles. **(B, C)** Cells grown in the presence of *C. copticum* at 90 μg.mL^−1^; loosening of cell membrane (1), disorganized cytoplasm (2), deposition of lipid globules (3), excessive vacuolization (4). **(D,E)** Cells grown in the presence of *T. vulgaris* at 90 μg.mL^−1^; disorganized protoplasm with receding of cell membrane (5), thickening of cell wall (6). **(F)** Cells grown in the presence of thymol at 90 μg.mL^−1^; lysis of cell wall and shrinkage of cell (7).

In untreated sample of *Candida* cells, organelles such as nuclei, mitochondria and nucleus are appeared to be normal (Figure 3A). Treated sample exhibited several changes including thickening of cell wall, stretching of cell membrane, leakage of cell wall and cell membrane, deposition of lipid globules, excessive vacuolization and abnormal distribution of polysaccharides leading to deterioration of cytoplasmic contents (Figure 3B-F).

## Discussion

Efficient treatment for fungal infections has become very important as the frequency of life-threatening fungal diseases is increasing due to the progress in the treatment of critically ill patients whose immune status has been deteriorated. Furthermore, high doses drug toxicity and emergence of drug-resistant strains have added extra burden for currently available antifungal therapy. Therefore, development of novel antifungal agents or strategies that are effective against pathogens that are resistant to currently available antifungal drugs is of paramount importance. Considering this, we attempted to investigate concentration dependent effect of two essential oils namely *C. copticum* and *T. vulgaris* and their major active compound thymol against growth and virulence factors production viz. proteinase and haemolysin and biofilm formation in the drug-resistant strains of *Candida* spp.

Our data has highlighted the production of proteinase and haemolysin in the test strains of *Candida* spp irrespective of their source of clinical conditions. Productions of these virulence factors are in agreement of reports of other workers [39–41]. Furthermore, hydrophobicity seems to be associated with biofilms forming ability of the strains. This could be a very serious threat for removal of infections caused by drug-resistant strains especially by conventional antifungal drugs such as amphotericin B and azoles because of their limitations due to host toxicity and drug-resistance [9]. These test oils were found to be non-toxic at sub-MICs. Thymol was the major active ingredient of both the test oils as revealed by GC/GC-MS analysis and found to be in agreement with the literature [16, 19, 20]. Moreover, thymol was found to be more effective in all the inhibitory assays. This indicates that inhibitory activity of the oils of *C. copticum* and *T. vulgaris* is largely due to the thymol. However, minor constituents may also play a key role in the biological activities of these oils.

In this study, all the oils at tested sub-MICs significantly reduced the CSH in test strains. Hydrophobic cells are more adherent and resistant to phagocytosis and, therefore more virulent than hydrophilic cells. In this regard, lowering of CSH by test oils suggests their importance in arresting the candidal colonization in pathogenesis. Furthermore, our findings have revealed strong inhibition of proteolytic activity by test oils at 0.25 × and 0.5 × MICs. Secreted aspartyl proteinases (SAPs) degrade many lesion proteins at lesion sites, such as albumin, hemoglobin, keratin, and secretory immunoglobulins A [42]. These proteolytic activities of SAPs are important for the virulence of *Candida* spp. Therefore, inhibition of extracellular production of proteinase will weaken the colonization of *Candida* cells and will be easily evaded by immune cells. Also, test oils effectively inhibited the haemolysin production at 0.25 × MIC. In our knowledge, there is no report on inhibition of haemolysin production in *C. albicans* by essential oils. There are reports that high concentrations of azole drugs cause an increased Sap activity in resistant isolates [43]. AMB-resistant strain developed from *C. albicans* ATCC10231 exhibited enhanced activity of virulence factors like extracellular secreted aspartyl proteinase [44]. In this perspective, oils of *C. copticum*, *T. vulgaris* and thymol exhibiting anti-virulence activity along with conventional antifungal drugs will be of great advantage in controlling the increase in virulence of drug-resistant strains. Drug-resistant strains exhibiting production of virulence factors will be more serious in case of immuno-compromised patients where relapses of infection occurs because high doses can not be given and weakened immune system could not clear the infection. The use of anti-virulence agent at lower doses of sub-inhibitory concentration will assist in combating such problems.

Furthermore, our data showed that 77% of *C. albicans* strains were strong biofilm formers. This may reflect similarities in the ability of strains of *C. albicans* from different sites of infection to form biofilms and strengthen the reports from other workers that most of the *Candida* infections are associated with biofilm forming ability of *Candida* spp. We evaluated oils of *C. copticum*, *T. vulgaris* and thymol at sub-MICs for their ability to inhibit the formation of biofilms. If an agent is added at the beginning of the experiment, the agent might act before the biofilm formed and inhibit development of biofilm; it could be of greater interest in combating recalcitrant infections of *Candida* biofilms. Our data revealed varying level of attenuation of biofilm formation by planktonic *Candida* cells in the presence of test oils and drugs in a dose dependent manner. Among the tested agents, thymol showed most inhibitory effect on biofilm formation at 0.5 × and 0.25 × MIC followed by *T. vulgaris* and *C. copticum*. Control drug fluconazole was less effective in preventing the formation of biofilms. The concentration dependent inhibition of biofilm formation was visually confirmed by scanning electron microscopy of *C. albicans* 04. In our study, SEM observations have revealed intact biofilm formation by untreated cells in 48 h whereas treated cells exhibited disorganization of biofilm stages. This is appeared to be because of interference of oils with cell membrane integrity as evidenced by shrinkage of cell surface and lysis of sessile cells. Similar observations were reported by Braga *et al.* [45] for thymol and eugenol against planktonic cells of *C. albicans*. This indicates that *Candida* cells in both the planktonic and biofilm stages are affected in a similar fashion and cells in biofilm stage can not gain increased tolerance to test oils as it attains against conventional antifuingal drugs. TEM observations of treated *Candida* cells indicate that mode of action of such compounds are disrupting the overall intracellular endomembranous system. Therefore, it appears that cell wall and cell membrane integrity, along with other membranous structures are the target sites of these compounds. The hydrophobic and volatile nature of oils may result in their increased uptake through charged polysaccharides of extracellular matrix of biofilms making more cells in contact to oils and exerting greater membrane permeation into *Candida* cells. This may result in an increased spectrum of action of these oils on sessile cells including persister cells leading to a retarded or disorganized development of biofilms.

## Conclusions

In conclusion, the non-growth inhibitory concentrations i.e. sub-MICs of oils of *C. copticum* and *T. vulgaris* could influence the production of proteinase and haemolysin and biofilm formation. These activities appeared to be due to the presence of thymol in these oils. Inhibition of production of proteinase and haemolysin factors and biofilms by these oils will add to their efficacy as anti-pathogenic agents. A potential advantage of this approach is that new antimicrobials aimed at inhibiting virulence rather than growth may impose weaker selective pressure for the development of antibiotic resistance relative to current antibiotics. Novel therapeutics that target virulence rather than simply *in vitro* cell growth would both supplement and add diversity to our current antimicrobial armamentarium However, pharmacokinetic studies are needed to ensure the toxicity and anti-pathogenic ability of these oils under *in vivo* to establish their therapeutic potential.
